# The application of quality control circle activities in the management of clinical undergraduate pediatric internship teaching

**DOI:** 10.1186/s12909-026-09055-4

**Published:** 2026-03-21

**Authors:** Wenjing Ou, Zhihui Liu, Meiling Cao, Chao Wu, Min Zhao

**Affiliations:** 1 Department of Medical Record Administration, Baotou Central Hospital, Baotou, Inner Mongolia 014040 China; 2 Department of Anesthesiology, Baotou Central Hospital, Baotou, Inner Mongolia 014040 China; 3 Department of Information Technology, Baotou Central Hospital, Baotou, Inner Mongolia 014040 China; 4 Department of Imaging Centre, Baotou Central Hospital, Baotou, Inner Mongolia 014040 China; 5 Department of Pediatrics, Baotou Central Hospital, No. 61, Huan Cheng Road, Donghe District, Baotou, Inner Mongolia 014040 China

**Keywords:** Quality Control Circle, Mini-CEX, DOPS, Pediatric Clinical Practice

## Abstract

Traditional pediatric internship training for undergraduate medical students is often limited by student passivity and underdevelopment of clinical reasoning and skills. This study aimed to evaluate the effectiveness of the Quality Control Circle (QCC) method, integrated with Mini-Clinical Evaluation Exams (Mini-CEX) and Direct Observation of Procedural Skills (DOPS) assessments, in enhancing clinical competencies and teaching outcomes during pediatric internships. Eighty-four medical students were randomly assigned to a QCC group (traditional teaching plus QCC activities) or a control group (traditional teaching only). Clinical skills were assessed using Mini-CEX and DOPS before and after the 4-week pediatric internship. Final exam results and teaching satisfaction were also compared. Baseline scores showed no significant differences. Post-intervention, both groups improved, but the QCC group demonstrated significantly greater improvement in all Mini-CEX domains (medical history taking, physical examination, professionalism, clinical judgment, communication, efficiency, overall competence) and all 11 DOPS items (e.g., procedural understanding, aseptic technique, communication) (*P* < 0.05). The QCC group also scored higher in final theoretical exams, skill operations, and medical record writing (*P* < 0.05). Self-evaluations indicated significant progress in eight comprehensive competencies, notably problem-solving and communication skills (*P* < 0.05). Teaching satisfaction was significantly higher in the QCC group (92.85%) than in the control group (76.19%) (*P* = 0.024). The QCC teaching model significantly enhances clinical skills, assessment performance, comprehensive competencies, and teaching satisfaction in pediatric internships compared to traditional methods, proving to be a valuable approach for improving clinical teaching quality.

**Supplementary Information:**

The online version contains supplementary material available at 10.1186/s12909-026-09055-4.

## Introduction

Pediatrics, as a vital component of clinical medical education, possesses unique characteristics that necessitate distinctive teaching approaches. Children are not miniature adults; Consequently, they exhibit significant differences in disease spectrum, diagnostic methods, medication use, and disease progression [1]. Furthermore, pediatrics is often termed the “silent discipline,” as patients’ limited expressive abilities make clinical judgment heavily reliant on physicians’ observational skills and comprehensive capabilities [1, 2]. Therefore, pediatric clinical rotations serve not only as a crucial stage for translating theoretical knowledge into practice but also as a pivotal phase for cultivating medical students’ clinical reasoning abilities, procedural skills, and humanistic care awareness. Establishing a scientific and systematic clinical competency assessment and feedback mechanism plays a decisive role in enhancing the effectiveness of these rotations and shaping high-caliber pediatric medical professionals [3].

Traditional pediatric clinical training predominantly employs teacher-centered lectures and demonstrations, leaving students in a passive receptive role. This approach lacks active participation and timely feedback, resulting in inadequate clinical reasoning training, unstable skill mastery, and weak communication abilities [4, 5]. To address the limitations of traditional assessment methods, Mini-Clinical Evaluation Exams (Mini-CEX) and Direct Observation of Procedural Skills (DOPS) have gained increasing recognition in recent years as formative assessment tools [6, 7]. Mini-CEX, introduced by the American College of Physicians in 1995, focuses on assessing comprehensive competencies including clinical communication, history-taking, physical examination, and diagnostic decision-making [8, 9]. Meanwhile, DOPS emphasizes direct observation and feedback on the standardization and proficiency of clinical procedural skills [10, 11]. The combination of these two tools comprehensively covers the “diagnosis-management” process and can be conveniently implemented across diverse settings such as outpatient clinics, emergency departments, and inpatient wards. Immediate feedback facilitates continuous improvement for medical students during practice, making this approach particularly suitable for pediatric clinical scenarios characterized by complex conditions and high procedural demands [12–14].

However, the sole introduction of Mini-CEX and DOPS still faces challenges such as inconsistent implementation quality, inefficient teaching effectiveness, and insufficient cultivation of team collaboration awareness [15]. Against this backdrop, Quality Control Circle (QCC)—a quality management approach originating from corporate management that emphasizes teamwork and continuous improvement [16]—offer new insights for medical education management. QCC forms groups comprising both faculty mentors and trainees. Through problem-oriented, collaborative brainstorming, they systematically analyze teaching challenges and propose improvement strategies, thereby promoting refinement, standardization, and efficiency in the teaching process [17]. While QCC have demonstrated positive outcomes in nursing education and clinical quality management [18, 19], their systematic application in managing clinical internships for undergraduate pediatric medical students warrants further exploration.

Therefore, this study proposes to integrate quality circles into the management of pediatric internship training for undergraduate medical students. By establishing a competency assessment feedback system based on Mini-CEX and DOPS, and combining it with the team collaboration and process optimization mechanisms of quality circles, we will systematically evaluate its effectiveness in enhancing medical students’ comprehensive clinical competencies and promoting mutual growth in teaching and learning.

## Methods

### Study population

84 clinical undergraduate students from Inner Mongolia Medical University, who were undergoing clinical internships at the affiliated hospital from October 2024 to September 2025, were selected as the study subjects. All participants were randomly divided into a QCC group (*n* = 42) and a control group (*n* = 42) using a random number table (Clinical trial number: not applicable). The internship in the pediatrics department lasted for four weeks. The QCC group established a QCC activity team, while the control group received traditional teaching.

#### Inclusion criteria

1.  The clinical medical students were full-time undergraduate students or above;
2. The clinical medical students were informed of the research purpose and protocol and participated voluntarily;
3.  The clinical medical students completed a four-week internship in the pediatrics department.

#### Exclusion criteria:

1. Students from secondary specialized schools, junior colleges, or health vocational institutions;
2.  Those who declined to participate or withdrew from the study;
3.  Those who withdrew or were unable to complete the four-week internship training;
4.  Those with prior relevant internship or learning experience;
5.  Those concurrently participating in other related studies.

### Teaching methods

All instructors involved in training and assessment were from the Department of Pediatrics at Baotou Clinical Hospital of Inner Mongolia Medical University. Holding lecturer-level or higher professional titles, they underwent standardized training to ensure consistent grading criteria, achieve homogeneous management, and minimize training and evaluation biases caused by individual differences.

The control group medical students received traditional clinical instruction delivered by five clinical physicians, comprising lectures, clinical practice, and post-class theoretical assignments. Theoretical teaching was uniformly conducted by instructors with periodic assessments. Practical training focused on simulation exercises supplemented with bedside teaching, where instructors explained disease-related knowledge and, with patient consent, guided students in hands-on procedures within the ward.

The QCC group incorporated the QCC teaching management model into traditional instruction, establishing a pediatric clinical internship QCC teaching team. The specific implementation plan is as follows.

### Organizational structure of QCC in pediatric clinical internship teaching

First, clinical instructors in the QCC group received systematic training on QCC knowledge and formed a QCC team. This team comprised five members: one circle leader and four circle members. The circle leader, a pediatric associate chief physician with 14 years of teaching experience, served as the primary responsible party. This role encompassed coordinating activity plans, drafting evaluation protocols, gathering feedback, overseeing teaching quality, and organizing implementation. The circle members consisted of four pediatric clinical teachers with intermediate professional titles, all holding a bachelor’s degree or above. They are responsible for providing feedback and coordination during the implementation of the QCC.

Incorporating the concept of quality circles into teaching design based on the characteristics of pediatric clinical education, we established the circle name and scheduled regular meetings. Prior to classes, medical students in the QCC group formed study teams, designed group names, and explained their meanings to enhance students’ sense of belonging and participation. The circle was named the “Pediatric Excellence Circle,” symbolizing the teaching team’s commitment to pursuing outstanding teaching quality and aiming to stimulate students’ autonomy, initiative, and creativity. Circle meetings were scheduled for Mondays from 16:00 to 17:00 in the Pediatric Department’s seventh-floor medical office, focusing on discussions to optimize and improve teaching methodologies.

### Goal setting

Through quality circle teaching management, students are enabled to solidly master fundamental theoretical knowledge after completing pediatric clinical internships, while enhancing their clinical skills, medical record writing, humanistic care, and doctor-patient communication abilities. Concurrently, this approach seeks to boost students’ learning interest, initiative, motivation, and overall satisfaction with the teaching process.

### Strategy development and implementation

**Fig. 1:**
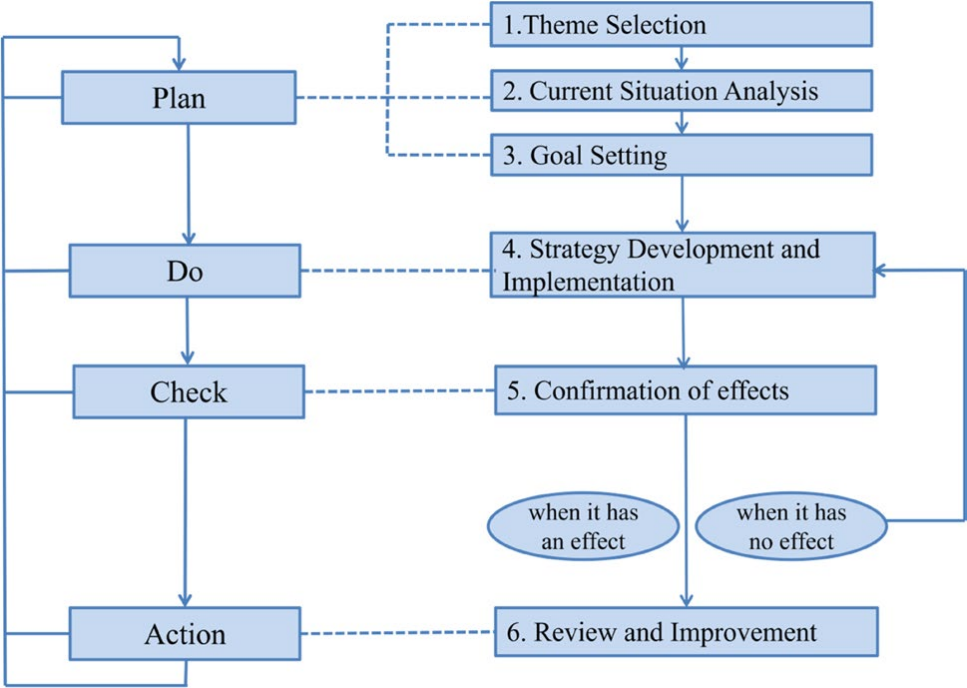
Basic steps of PDCA cycle and QCC activities. Quality Control Circle is a management method that solves practical problems through team collaboration and scientific procedures, with its core being the achievement of quality improvement through cyclical refinement

Based on problem analysis outcomes, targeted strategies were developed and implemented. Classroom instruction was designed according to the Plan-Do-Check-Act (PDCA) cycle, a four-step management model for continuous improvement that involves planning an intervention, implementing it, evaluating the results, and taking corrective action based on the findings. This framework integrated diverse teaching methodologies including project-based learning, group discussion, demonstration-feedback-evaluation-redemonstration cycles, and problem-oriented teaching (see Fig. 1). Meanwhile, supervision and management of the teaching implementation process were strengthened. Teaching inspections were conducted to standardize the instructors’ teaching procedures, ensuring that they strictly followed the established teaching methods during lessons, thereby safeguarding the quality of both theoretical and practical courses.

In this study’s teaching practice, the QCC group implemented the QCC teaching method: moving away from traditional didactic teaching, establishing a student-centered teaching philosophy, and stimulating students’ learning initiative. Students were divided into learning groups of 6–8, each with a group leader, encouraging them to take an active role within the group and collaboratively complete tasks assigned by the teacher. During training in key procedures such as lumbar puncture, bone marrow aspiration, physical measurements, and endotracheal intubation, students were guided to proactively identify problems and analyze causes using brainstorming techniques. Based on this, teachers developed targeted teaching countermeasures. If deficiencies were identified in the teaching process, teaching priorities and strategies were promptly adjusted, and remedial teaching was provided to address students’ individual needs. Teaching methods such as case analysis and role-playing were introduced to guide students in independently discovering and solving problems in real-life situations, deepening their understanding of knowledge. Teachers regularly evaluated learning outcomes.

Concurrently, efforts were made to enhance training in pediatric clinical skills and medical record writing, moving beyond the traditional theory-focused approach. This change effectively strengthened students’ practical abilities. Throughout the management of medical students, instructors consistently prioritized the development of comprehensive practical skills, focusing on the coordinated advancement of clinical procedures, theoretical knowledge, patient observation, and doctor-patient communication. The entire teaching process strictly adhered to the PDCA cycle principle, integrating multiple methods such as project-based teaching, group discussions, demonstration-feedback-redemonstration cycles, and problem-oriented teaching. Teaching strategies were continuously optimized based on students’ actual learning needs, and teachers were required to continually reflect on and improve their teaching methods from the students’ perspective, thereby achieving steady enhancement of teaching quality.

### Effectiveness evaluation indicators

Two standardized assessment tools were employed to evaluate the clinical competencies of pediatric medical students: the Mini-CEX [20] and DOPS [20]. The Mini-CEX evaluates seven key areas in a phased manner: medical history taking, physical examination, medical ethics, clinical diagnosis/treatment plan, doctor-patient communication, organizational effectiveness, and overall evaluation. The DOPS evaluates the following aspects: understanding of clinical skill indications and procedures; ability to provide detailed patient information and obtain informed consent; pre-procedure preparation; administration of appropriate analgesia and sedation; technical proficiency in performing clinical procedures; aseptic technique; ability to seek assistance when needed; post-procedure management; communication skills with patients; consideration of patient feelings and professional standards; and overall performance in executing clinical skills. The detailed scoring rubrics for both tools are provided as supplementary material.

### Components of the departmental exit examination

For medical students completing their rotations during the same period, the theoretical exam is based on the same set of questions randomly selected from the department exit exam question bank. The clinical skills assessment involves one randomly selected skill from the skills assessment question bank. Medical record writing is evaluated with reference to the “Intern Medical Student Inpatient Medical Record Quality Evaluation Form,” and the ideological and moral assessment is conducted using the “Clinical Undergraduate Student Ideological and Moral Assessment Score Sheet” (see Supplementary Material 2, 3). The final departmental exam score is calculated as follows: theoretical exam (30%), clinical skills assessment (40%), medical record writing (20%), and ideological and moral assessment (10%).

### Intangible outcomes assessment

Intangible outcomes refer to the non-quantifiable, indirect benefits of the intervention. These were assessed by quantitatively analyzing the self-evaluation scores provided by QCC members upon completion of the QCC activity. Radar charts were utilized to provide a visual comparison of the intervention’s impact across eight dimensions: problem-solving ability, communication skills, sense of responsibility, team cohesion, mastery of quality control methods, innovative capabilities, proactive attitude, and professional knowledge.

### Teaching satisfaction

Upon completion of clinical rotations, both groups of medical students were required to complete a teaching satisfaction questionnaire [21]. The survey contained a single question regarding overall teaching satisfaction, with five response options: Very Dissatisfied, Dissatisfied, Neutral, Satisfied, and Very Satisfied. Medical students anonymously completed the questionnaires, which were collected on-site by the teaching secretary for statistical analysis. Teaching Satisfaction Rate (%) = (Number of Satisfied + Number of Very Satisfied) / Total Number of Respondents in the Group × 100%.

### Evaluation protocol

Prior to and following their pediatric rotation, two physicians at the level of attending physician or above, who had received specialized training, scored and evaluated the medical students from both groups.

### Statistical methods

All data were processed using SPSS 24.0 statistical software. Count data are expressed as rates and compared using chi-square tests. Continuous variables are presented as mean ± standard deviation or median (interquartile range) and compared using independent samples t-tests or nonparametric rank sum tests. *P* < 0.05 was considered statistically significant.

## Results

### Comparison of baseline data

A total of 84 undergraduate medical students majoring in clinical medicine who underwent clinical training in the pediatric department of our hospital from October 2024 to June 2025 were enrolled as study subjects. Prior to clinical training, they were randomly assigned using a random number table to either the traditional teaching group or the QCC group, with 42 students in each group. There were no statistically significant differences in gender or age between the two groups (*P* > 0.05), indicating that the groups were comparable. See Table 1.

**Table 1:** Basic Information of the two groups of students

Group	Age(‾x±s)	Gender(*n*,%)	
		Male	Female
Control Group (*n* = 42)	23.98 ± 0.75	25(59.50)	17(40.50)
Quality Control Circle Group (*n* = 42)	23.83 ± 0.91	18(42.90)	24(57.10)
t/χ^2^	0.787	2.335	
*P*	0.434	0.127	

### Comparison of mini-CEX scores between groups

Prior to clinical training, there were no statistically significant differences between the two groups in the seven Mini-CEX scores (*P* > 0.05). After clinical practice, both the control group and the quality circle group showed increased scores in all seven items: medical record collection, physical examination, medical ethics, clinical diagnosis/treatment plan, doctor-patient communication, organizational effectiveness, and overall evaluation. Furthermore, all scores in the QCC group were higher than those in the control group (*P* < 0.05), as shown in Table 2.

**Table 2:** Comparison of mini-CEX scores between pre- and post-training groups (Scores, ‾x±s)

Time	Group	Medical History Collection	Physical Examination	Medical Ethics	Clinical Diagnosis/Treatment Plan	Doctor-Patient Communication	Organizational Effectiveness	Overall Evaluation
Pre-Training	Control Group (*n* = 42)	4.91 ± 0.66	4.26 ± 0.70	5.12 ± 0.89	4.83 ± 0.82	5.19 ± 0.89	4.71 ± 0.86	5.26 ± 0.80
	Quality Control Circle Group (*n* = 42)	4.86 ± 0.75	4.21 ± 0.89	5.07 ± 0.89	4.81 ± 0.83	5.07 ± 0.92	4.69 ± 0.84	5.05 ± 0.83
	t	0.31	0.333	0.245	0.132	0.602	0.128	1.21
	*P*	0.758	0.74	0.807	0.896	0.549	0.898	0.23
Post-Training	Control Group (*n* = 42)	6.05 ± 0.76	5.81 ± 0.55	5.91 ± 0.85	5.93 ± 0.78	5.98 ± 0.81	5.98 ± 0.72	5.81 ± 0.92
	Quality Control Circle Group (*n* = 42)	7.36 ± 0.49	7.31 ± 0.47	7.45 ± 0.50	7.33 ± 0.48	7.43 ± 0.50	7.40 ± 0.50	7.45 ± 0.50
	t	-9.382	-13.439	-10.151	-9.98	-9.873	-10.631	-10.176
	*P*	0.000	0.000	0.000	0.000	0.000	0.000	0.000

### Comparison of DOPS scores between groups

Prior to clinical practice and training, the 11 DOPS scale scores showed no statistically significant differences between the two groups (*P* > 0.05). After clinical practice, the control group and QCC group both demonstrated increased scores in 11 items: understanding of clinical skill indications and procedures, informing patients and obtaining consent, pre-procedure preparation, appropriate comfort and pain relief, procedural skills, sterility awareness, seeking assistance when needed, post-procedure management, communication skills with patients, demonstration of professionalism/humanistic care, and overall performance. All scores in the QCC group were higher than those in the control group (*P* < 0.05), as shown in Table 3.

**Table 3:** Comparison of DOPS scores between pre- and post-training groups (scores, ‾x±s)

Time	Group	Understanding the indications and procedures for clinical skill operations	Inform the patient and obtain consent	Preparation before the procedure	Appropriate reassurance and pain relief	Operational skills	Sterility principles	Seek assistance as needed	Post-procedure management	Communication techniques with patients	Demonstrate professionalism/humanistic care	Overall performance
Pre-Training	Control Group (*n* = 42)	5.74 ± 0.49	5.76 ± 0.48	5.69 ± 0.52	5.21 ± 0.52	5.21 ± 0.75	5.43 ± 0.55	5.71 ± 0.64	5.45 ± 0.77	5.52 ± 0.71	5.74 ± 0.45	5.33 ± 0.61
	Quality Control Circle Group (*n* = 42)	5.79 ± 0.52	5.74 ± 0.54	5.48 ± 0.80	5.24 ± 0.48	5.36 ± 0.73	5.43 ± 0.63	5.74 ± 0.13	5.31 ± 0.12	5.48 ± 0.67	5.62 ± 0.66	5.26 ± 0.59
	t	-0.429	0.212	1.453	-0.217	-0.887	0	-0.148	0.861	0.317	0.968	-0.546
	*P*	0.669	0.833	0.15	0.829	0.378	1	0.883	0.392	0.752	0.336	0.586
Post-Training	Control Group (*n* = 42)	6.69 ± 0.60	6.74 ± 0.54	6.55 ± 0.59	6.43 ± 0.67	6.41 ± 0.66	6.50 ± 0.59	6.74 ± 0.70	6.69 ± 0.56	6.67 ± 0.65	6.74 ± 0.59	7 ± 1.01
	Quality Control Circle Group (*n* = 42)	7.12 ± 0.33	7.05 ± 0.22	7.10 ± 0.30	7.07 ± 0.26	7.43 ± 0.50	7.14 ± 0.47	7.19 ± 0.45	7.05 ± 0.38	7.05 ± 0.38	7.45 ± 0.50	7.43 ± 0.63
	t	-4.04	-3.43	-5.353	-5.811	-7.972	-5.486	-3.511	-3.411	-3.279	-5.985	-2.329
	*P*	0.000	0.001	0.000	0.000	0.000	0.000	0.001	0.001	0.002	0.000	0.022

### Comparison of departmental exit examination scores between the two groups

The QCC group scored significantly higher than the control group in theoretical knowledge, skill operations, medical record writing, and final departmental exam scores (*P* < 0.05). However, no statistically significant difference was observed between the two groups in the assessment of ideological and moral character, as shown in Table 4.

**Table 4:** Comparison of Theoretical Knowledge, Skill Operations, Medical Record Writing, and Ideological and Moral Character in the Final Departmental Examinations for Two Groups of Medical Students (Scores, ‾x±s)

Group	Theoretical Knowledge	Skill Operations	Medical Record Writing	Ideological and Moral Character	Departmental Exam Scores
Control Group (*n* = 42)	22.15 ± 2.50	29.47 ± 5.35	14.43 ± 3.59	9.28 ± 0.64	77.83 ± 4.15
Quality Control Circle Group (*n* = 42)	24.50 ± 1.79	32.43 ± 2.37	16.45 ± 2.38	9.45 ± 0.55	86.91 ± 2.38
t	-5.994	-3.278	-3.048	-1.285	-12.295
*P*	0.000	0.002	0.003	0.203	0.000

### Comparison of intangible outcomes among QCC group students before and after QCC implementation

**Fig. 2:**
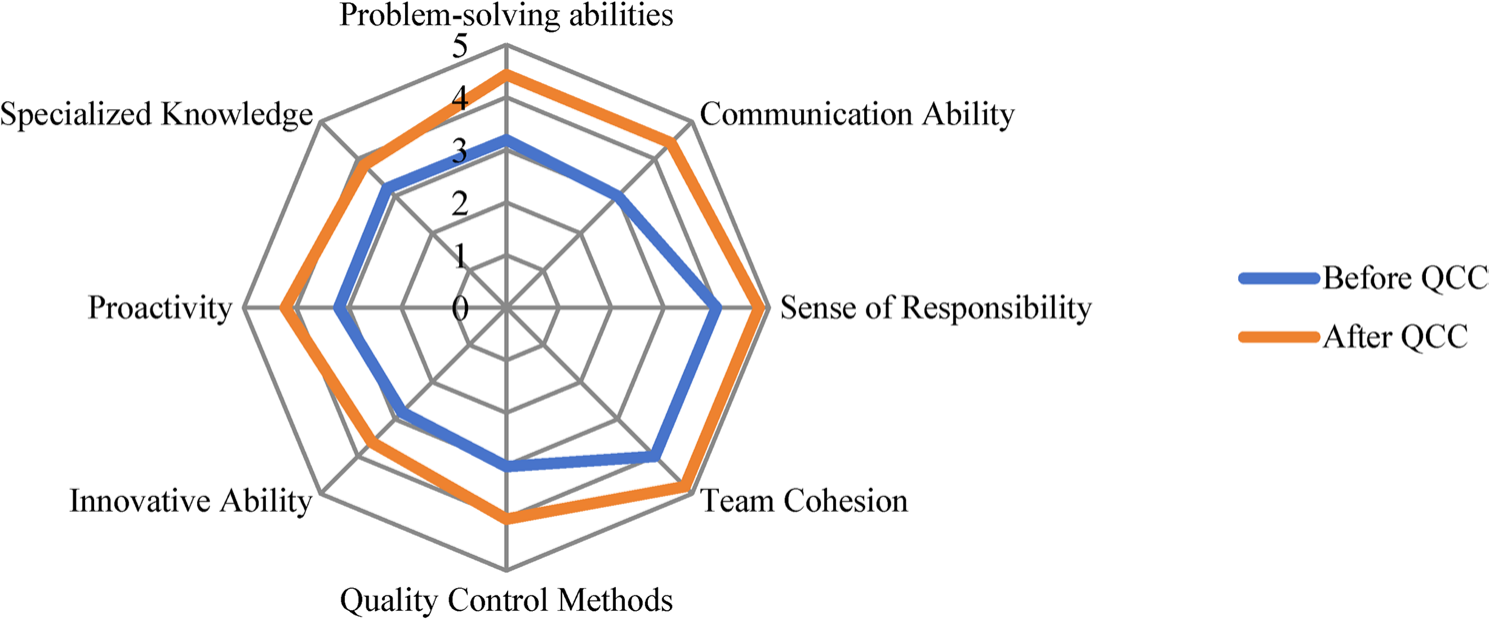
Intangible results before and after the implementation of QCC activities

Following QCC implementation, QCC group students were assessed across eight key skill domains: problem-solving, communication skills, sense of responsibility, team cohesion, quality management methods, innovation capability, proactivity attitude, and professional knowledge. Notably, students in the QCC group scored significantly higher in all eight domains post-intervention (*P* < 0.05), with particularly notable progress in communication skills, problem-solving ability, quality management methods, and proactive attitude. Self-assessments of students before and after QCC implementation are shown in Table 5; Fig. 2.

**Table 5:** Self-assessment scores of QCC group students before and after quality control circle implementation

Evaluation Criteria	Before QCC			After QCC			Activity Growth +/–	Growth Rate	t	*P*
	Total Score	Average Score	Standard Deviation	Total Score	Average Score	Standard Deviation				
Problem-Solving Ability	134	3.19	0.40	186	4.43***	0.50	1.24	37.5	-12.549	0.000
Communication Skills	126	3	0	186	4.43***	0.50	1.43	46.66	-18.484	0.000
Sense of Responsibility	168	4	0	202	4.81***	0.40	0.81	20	-13.2	0.000
Team Cohesion	168	4	0	202	4.81***	0.40	0.81	20	-13.2	0.000
Quality Control Methods	127	3.02	0.64	169	4.02***	0.64	1	33.33	-7.122	0.000
Innovative Capabilities	118	2.81	0.40	152	3.62***	0.49	0.81	28.57	-8.3	0.000
Proactive Attitude	134	3.19	0.40	177	4.21***	0.42	1.02	31.25	-11.543	0.000
Professional Knowledge	135	3.21	0.75	161	3.83***	0.76	0.62	18.75	-3.751	0.000

The maximum score for each item is 5 points per person, while the minimum score is 1 point (5 = very good, 4 = good, 3 = fair, 2 = poor, 1 = very poor). The total score for each evaluation item is the sum of the scores assigned by all circle members, with a maximum possible total of 55 points

The growth rate is calculated as follows: Growth Rate = (Average Score After QCC-Average Score Before QCC)/Average Score Before QCC)×100%

A higher growth rate indicates a greater improvement in the evaluation metrics among circle members

**P < 0.01, ***P < 0.001

### Comparison of overall teaching satisfaction results between the two groups

Regarding overall teaching satisfaction, the satisfaction rate among students in the QCC group was significantly higher than that in the control group (92.85% vs. 76.19%, *P* = 0.024). See Table 6 for details.

**Table 6:** Student satisfaction with overall teaching

Group	Very Satisfied	Satisfied	Neutral	Dissatisfied	Satisfaction Rate (%)
Control Group (*n* = 42)	17(40.50)	15(35.70)	5(11.90)	5(11.90)	76.19
Quality Control Circle Group (*n* = 42)	30(71.40)	9(21.40)	1(2.40)	2(4.80)	92.85
χ^2^	9.398				
*P*	0.024				

## Discussion

In this teaching study involving 84 pediatric medical students at our hospital, we systematically compared the combined effects of traditional teaching methods and the QCC teaching model. Through multidimensional data including Mini-CEX assessments, DOPS evaluations, departmental exit examinations, and intangible outcome assessments, this study demonstrates that the QCC teaching method demonstrates significant advantages in enhancing the comprehensive competencies of undergraduate medical students. Specifically, in terms of clinical competency development, the QCC group scored significantly higher than the traditional teaching group across all seven core competencies on the Mini-CEX scale and all eleven procedural skills on the DOPS scale. In the departmental exit assessments, the QCC group achieved higher scores in all three objective measures: theoretical exams, procedural skills, and medical record documentation. Notably, after QCC implementation, students demonstrated significant improvements across eight intangible dimensions, including problem-solving and teamwork. Furthermore, the QCC group reported overall teaching satisfaction at 92.85%, significantly higher than the traditional group’s 76.19%. Overall, this study combines quantitative data with subjective evaluation metrics to preliminarily validate that QCC teaching activities not only effectively enhance clinical medical students’ professional competence and skill levels but also play a significant role in cultivating non-cognitive competencies and improving teaching satisfaction. This provides empirical evidence for clinical teaching reform.

### Enhancement of clinical competence through QCC teaching method in mini-CEX and DOPS assessments

This study systematically evaluated the effectiveness of the QCC teaching method in clinical medical internship education, focusing on its role in enhancing students’ clinical competence as measured by Mini-CEX and DOPS assessments.

In the Mini-CEX assessment, medical students in the QCC teaching group demonstrated significant improvements across multiple dimensions, ranging from technical skills like medical history taking to non-technical skills such as teamwork. This comprehensive progress confirms that the QCC teaching method, through its core mechanisms of teamwork, problem orientation, and continuous improvement, can create an integrated learning experience, thereby promoting the synergistic development of students’ comprehensive clinical competencies. Previous research has indicated that effective clinical teaching requires not only the transmission of knowledge and skills but also the cultivation of students’ comprehensive adaptive capabilities in complex clinical environments [22]. The multi-dimensional enhancement effect of the QCC teaching method precisely addresses this educational need. It is worth noting that the Mini-CEX assessment tool employed in this study has been demonstrated in prior research to possess good reliability and validity [23, 24], which provides a solid foundation for the credibility of our findings.

In terms of clinical skills, the QCC teaching group performed significantly better than the traditional teaching group across all eleven DOPS evaluation indicators (*P* < 0.05). As a standardized tool for assessing procedural skills, DOPS provides a systematic framework of evaluation dimensions—including mastery of indications, procedural steps, preoperative preparation, aseptic technique, communication skills, and patient-centered care—that facilitates objective comparison of the effectiveness of different teaching methods. The advantage of the QCC teaching method lies in its ability to promote a shift from passive reception to active inquiry in students’ procedural skill learning through processes such as group discussion, cause analysis, and countermeasure implementation. This aligns closely with the critical role of “reflective practice” in skill acquisition emphasized in existing research [25]. In contrast, under the traditional teaching model, students often passively receive knowledge and lack systematic feedback and improvement mechanisms, which to some extent constrains the enhancement of teaching effectiveness.

Notably, although students in the traditional teaching group also demonstrated improvements in various competencies after the introduction of standardized assessment tools—existing research has confirmed that structured assessment tools themselves can enhance learning outcomes by providing clear criteria and immediate feedback [2, 26]—the QCC teaching group achieved more significant progress. This finding suggests that the value of the QCC teaching method lies not merely in its simple combination with assessment tools, but rather in its capacity to deeply integrate assessment feedback into the learning process through mechanisms of teamwork and iterative improvement, thereby amplifying the educational effectiveness of the assessment tools. As medical education continues to evolve, the integration of assessment tools with teaching methodologies has emerged as a critical issue in enhancing clinical teaching effectiveness [27, 28].

Pediatrics, as a highly specialized clinical discipline, is characterized by difficult patient communication and high procedural demands, which impose greater requirements on the comprehensive competencies of medical students. Previous studies have shown that the Mini-Clinical Evaluation Exercise (Mini-CEX), through structured assessment and immediate feedback, has unique value in enhancing students’ core clinical abilities [9], and is particularly suitable for evaluating comprehensive clinical qualities such as communication skills with pediatric patients’ families and professional conduct. The Direct Observation of Procedural Skills (DOPS), on the other hand, addresses the limitations of Mini-CEX in assessing procedural skills by directly observing and providing feedback on operational performance [10]. The innovation of this study lies in its integration of the QCC teaching method with these two standardized assessment tools, fully leveraging the strengths of the QCC approach in integrating multiple evaluations and promoting continuous improvement. The structured problem-solving process of QCC provides students with a platform to transform feedback from Mini-CEX and DOPS into concrete improvement actions. This “assessment-feedback-improvement” closed-loop mechanism may be a key factor contributing to its superior teaching effectiveness.

In summary, the QCC teaching method demonstrates superior outcomes compared to traditional approaches in both Mini-CEX and DOPS evaluations. It exhibits particularly significant advantages in enhancing students’ clinical competence and procedural skills, warranting further promotion and application in clinical medical internship education.

### Enhancement of medical students’ intangible outcomes before and after implementing the QCC teaching method

The QCC teaching method facilitates the development of core competencies essential for clinical practice, including problem-solving, communication, teamwork, and learning initiative.

Through QCC activities, students formed small learning teams. Active interactions during task division, collaborative learning, and problem discussions effectively broadened their thinking patterns, shifting knowledge acquisition from traditional passive reception to proactive exploration. This model enhances students’ ability to identify and resolve practical problems while boosting their learning motivation. It further stimulates their professional identity and enthusiasm, thereby promoting comprehensive personal development and demonstrating high educational applicability. Studies have shown that QCC activities significantly enhance participants’ problem-solving abilities and team cohesion [29], while also improving students’ teamwork awareness, communication skills, sense of responsibility, and professional identity in educational settings [30].

This research confirms that the QCC teaching method not only quantitatively enhances students’ clinical competencies in practice but also systematically promotes the comprehensive improvement of their overall quality and professional proficiency through structured QCC activities.

### QCC teaching method enhances overall teaching satisfaction

The integration of the QCC model into clinical teaching yields notable improvements in the educational experience, particularly in terms of teaching satisfaction. By providing systematic QCC training and maintaining rigorous standards for clinical instructors, this approach enhanced their overall competence and teaching quality. This, in turn, enabled medical students to acquire more clinical knowledge and practical skills, resulting in higher satisfaction with instruction. The recognition from medical students served to further motivate instructors, thereby creating a virtuous cycle of improvement [31]. This finding is consistent with international research, which indicates that effective teaching behaviors by clinical instructors enhance residents’ self-efficacy and clinical competence while simultaneously improving learning satisfaction, ultimately optimizing the clinical learning environment and ensuring educational quality [32, 33].

### QCC teaching method makes the departmental exit exam scores more outstanding

The QCC group’s superior performance across all departmental exit examination components—theoretical knowledge, professional skills, and medical record writing—highlights the comprehensive educational advantages of this approach. By fostering learning interest and initiative through collaborative problem-solving, the QCC method enables medical students to acquire knowledge and skills more efficiently. This collaborative dynamic not only reinforces theoretical foundations but also enhances procedural proficiency and improves the quality of medical record documentation.

QCC activities emphasize full participation, forming continuous quality improvement teams based on voluntary principles. This approach enables the continuous optimization of teaching models through practical mentoring, steadily improving teaching standards, and ensuring sustainable teaching outcomes.

This study retains several limitations. First, all samples originated from a single tertiary teaching hospital, and the limited number of medical students included resulted in a small sample size, potentially restricting the generalizability and persuasiveness of the conclusions. The effectiveness of teaching practices requires objective validation based on larger samples. Future research aims to expand sample sizes to enhance the credibility and representativeness of results. Second, this study only evaluated the short-term effects of the QCC teaching method over a 4-week period in pediatric clinical internships, without examining its long-term impact. Future research will develop a systematic teaching model and conduct continuous tracking of its long-term educational outcomes. Additionally, the QCC teaching method is currently implemented only in pediatric internship training at our hospital and has not been extended to other specialties. Furthermore, the limited frequency of using the assessment scale in internship evaluations may affect the comprehensiveness of the results. Future long-term effect evaluations will increase assessment frequency and explore the feasibility of extending this method to more clinical specialties. Our research team will also continue to explore other feasible and efficient teaching innovation pathways to continuously enhance the quality and standards of pediatric clinical internship training.

## Conclusions

The findings of this study indicate that applying the QCC teaching method in pediatric clinical internships offers significant advantages over traditional teaching approaches. This method not only effectively enhances students’ scores in the seven Mini-CEX clinical competencies and eleven DOPS procedural skills, but also yields superior performance in the final departmental assessment, including theoretical knowledge, procedural skills, and medical record documentation. More importantly, the QCC teaching method significantly promotes the development of students’ comprehensive competencies, including problem-solving, communication and collaboration, and innovative thinking, with a teaching satisfaction rate of 92.85%. Therefore, QCC is an effective teaching model that comprehensively cultivates medical students’ clinical practice abilities and professional competencies, warranting its promotion and application in clinical medical education.

## Supplementary Information

Supplementary Material 1.

Supplementary Material 2.

Supplementary Material 3.

## Data Availability

The datasets generated and/or analyzed in this study are not publicly available. However, they are available from the corresponding author upon reasonable request.
