# Supplementary material for: The application of quality control circle activities in the management of clinical undergraduate pediatric internship teaching
Source: BMC Med Educ. 2026 Mar 21;26:692. doi: 10.1186/s12909-026-09055-4 (PMC13127043; doi:10.1186/s12909-026-09055-4)
Supplement: Supplementary file 1 — Supplementary Material 1. [file 12909_2026_9055_MOESM1_ESM.docx]

****1. Mini-CEX Assessment****
The Mini-CEX assessment scale was specifically designed for pediatric medical students [20]. This tool evaluates students across seven key clinical competencies, including medical history taking, physical examination, medical ethics, clinical diagnosis and treatment planning, doctor-patient communication, organizational efficiency, and overall clinical performance. Each competency is rated on a three-level, nine-point scale: scores of 4–6 indicate meeting expected standards, while scores of 7–9 represent excellence. Each assessment session lasted approximately 15 minutes, followed by a 10-minute feedback period.

****2. DOPS Assessment****
The DOPS assessment system was utilized to evaluate students' procedural skills across 11 aspects [20], including: understanding of clinical skill indications and procedures; ability to provide detailed patient information and obtain informed consent; pre-procedure preparation; administration of appropriate analgesia and sedation; technical proficiency in performing clinical procedures; aseptic technique; ability to seek assistance when needed; post-procedure management; communication skills with patients; consideration of patient feelings and professional standards; and overall performance in executing clinical skills. Each aspect is evaluated using the same three-level, nine-point scale: scores of 1–3 indicate below standard; 4–6 indicate meets standard; and 7–9 indicate excellence. Similar to the Mini-CEX, assessments lasted 15 minutes, followed by 10 minutes of structured feedback.
