# Supplementary material for: The application of quality control circle activities in the management of clinical undergraduate pediatric internship teaching
Source: BMC Med Educ. 2026 Mar 21;26:692. doi: 10.1186/s12909-026-09055-4 (PMC13127043; doi:10.1186/s12909-026-09055-4)
Supplement: Supplementary file 2 — Supplementary Material 2. [file 12909_2026_9055_MOESM2_ESM.docx]

****Clinical Undergraduate Student Ideological and Moral Assessment Score Sheet****

| Student ID |  | Name |  | School |  |
| --- | --- | --- | --- | --- | --- |
| Major |  | Grade |  | Assessment Time |  |
| Teaching and Research Section | | | |  | |
| Evaluation Indicators | | | | Points | Score |
| Professional Dedication and Work Responsibility | | | | 20 |  |
| Medical Practice Style and Scientific Approach | | | | 20 |  |
| Medical Ethics and Service Attitude | | | | 20 |  |
| Teamwork and Interpersonal Relationships | | | | 20 |  |
| Observance of Discipline and Labor Discipline | | | | 20 |  |
| Total Score | | | | 100 |  |
| Evaluation Opinions of the Teaching and Research Section:  Signature of Teaching and Research Section Director (Secretary):  Year Month Day | | | | | |

Note: The total score multiplied by 10% constitutes the final evaluation score for this item, with a maximum of 10 points (retain one decimal place, round half up).
