# Supplementary material for: The application of quality control circle activities in the management of clinical undergraduate pediatric internship teaching
Source: BMC Med Educ. 2026 Mar 21;26:692. doi: 10.1186/s12909-026-09055-4 (PMC13127043; doi:10.1186/s12909-026-09055-4)
Supplement: Supplementary file 3 — Supplementary Material 3. [file 12909_2026_9055_MOESM3_ESM.docx]

****Medical Students Inpatient Medical Record Quality Evaluation Form****

Hospital___________________ Department________ Student Name________ Class__________ Total Score______ Date______

Patient Name: Inpatient Medical Record No.: Diagnosis:

| Evaluation Content | Scoring Criteria | Full Score | Actual Score | Brief Comments |
| --- | --- | --- | --- | --- |
| Chief Complaint | 1. Errors in main symptoms or onset time 2. Omission of main symptoms or onset time 3. Chief complaint description does not meet requirements | 1. Deduct 1-2 points 2. Deduct 1-2 points 3. Score 0 points | 5 |  |
| History of Present Illness | 1. Unclear description of disease onset 2. Unclear or disorganized sequence of illness progression, or omissions 3. Unclear characteristics of main symptoms 4. Unclear accompanying symptoms 5. Unclear symptoms relevant for differential diagnosis or important negative symptoms 6. Incomplete description of treatment received 7. General symptoms not described | 1. Deduct 1-2 points 2. Deduct 1-2 points 3. Deduct 1-3 points 4. Deduct 1-2 points 5. Deduct 1-2 points 6. Deduct 1-2 points 7. Deduct 1-2 points | 15 |  |
| Other History | 1. Omitted items 2. Relevant negative history not mentioned 3. Incorrect order | 1. Deduct 1-2 points 2. Deduct 1-2 points 3. Deduct 1 point | 5 |  |
| Physical Examination | 1. Omitted items 2. Omission of important positive or negative signs 3. Incorrect order 4. Incorrect results 5. Incomplete or inaccurate description of important signs | 1. Deduct 1-2 points 2. Deduct 1-3 points 3. Deduct 1 point 4. Deduct 1-5 points 5. Deduct 1-4 points | 15 |  |
| Auxiliary Examinations | Omission of necessary auxiliary examinations, deduct 1 point per item |  | 5 |  |
| Diagnosis | 1. Error in or significant omission of primary diagnosis 2. Error in or omission of secondary diagnosis 3. Incorrect order | 1. Deduct 5 points 2. Deduct 1-3 points 3. Deduct 1-2 points | 10 |  |
| Case Summary | 1. Omission of one item among: main admission symptoms (cause) and time, general condition, important past history, positive signs, main auxiliary examination results - deduct 1-2 points per item 2. Summary too lengthy, too brief, or poorly written - deduct 1-2 points |  | 10 |  |
| Basis for Diagnosis | 1. Insufficient basis for diagnosis 2. Listing textbook content, lacking specific analysis of the patient's actual situation | Deduct 2-5 points Deduct 2-5 points | 10 |  |
| Differential Diagnosis | 1. Omission of necessary differential diagnoses 2. Lack of basis or method for differentiation | Deduct 2-5 points Deduct 2-5 points | 10 |  |
| Treatment Plan | 1. Errors 2. Omissions 3. Not specific | Deduct 2-5 points Deduct 2-5 points Score 0 points | 10 |  |
| Specialist Examination Findings | For each omitted item | Deduct 2-3 points | 5 |  |
| Format & Writing* | 1. Non-compliance with format requirements 2. Non-compliance with medical terminology 3. Each incorrect/misspelled character | (Note: When these non-compliant items exist, corresponding points should be deducted from the total score of the preceding 11 categories) | Deduct 1-2 points Deduct 1-2 points Deduct 0.5 points | Points to Deduct |
| ****Total Score**** |  |  | ****100**** |  |

Note: The Total Score multiplied by 20% constitutes the final evaluation score for this item, with a maximum of 20 points (retain one decimal place, round half up).
